# An Impact of Moisture on Thermal State of Flax and Hemp Shives Thermal Insulations

**DOI:** 10.3390/ma19020440

**Published:** 2026-01-22

**Authors:** Piotr Kosiński, Lidia Kwiatkowska, Agata Gorząch, Monika Kwiatkowska, Przemysław Brzyski

**Affiliations:** 1 Faculty of Geoengineering, University of Warmia and Mazury in Olsztyn, Heweliusza 10 Str., 10-724 Olsztyn, Poland; lidia.kwiatkowska@uwm.edu.pl (L.K.); agata.gorzach@student.uwm.edu.pl (A.G.); monika.kobylska@student.uwm.edu.pl (M.K.); 2 Faculty of Civil Engineering and Architecture, Lublin University of Technology, 40 Nadbystrzycka Str., 20-618 Lublin, Poland; p.brzyski@pollub.pl

**Keywords:** hemp shives, flax shives, moisture content, thermal conductivity, bulk density

## Abstract

Plant-based materials exhibit different moisture absorption properties than synthetic materials. In the case of synthetic fibrous insulation, the effect of moisture on thermal conductivity can be relatively easily determined based on the mass fraction of moisture in the material’s skeleton. In the case of cellulosic materials with an open capillary structure, determining this effect requires laboratory testing. The authors conducted laboratory tests of the thermal conductivity coefficient of dry and wet plant-based insulation, such as flax and hemp shives. The effect of material densification at various moisture levels was also considered. The article also presents a numerical analysis of the thermal state and moisture content of thermal insulation used in walls operating under moderate climatic conditions. For damp shives, thermal conductivity increases noticeably with increasing densification, while for dry shives, thermal conductivity decreases until a certain level of densification is achieved. The obtained results were compared with values calculated using a linear model of the relationship between thermal conductivity and moisture content in the material. At higher moisture values, around 14–15 wt.%, thermal conductivity results are significantly lower than those obtained from the linear model (12.5–16.3% in the case of flax shives and 8.4–11.3% in the case of hemp shives) This is a favorable characteristic of shives compared to the performance of, for example, mineral wool in elevated humidity conditions. The authors believe that their results will be not only scientific but also practical, facilitating the assessment of heat loss in buildings.

## 1. Introduction

Flax shives are obtained from the stalks of common flax (*Linum usitatissimum* L.), which is cultivated for its fiber and oil. Hemp shives, on the other hand, are obtained from the stalks of industrial hemp (*Cannabis sativa* L.), a modified plant species with reduced THC content that is typically cultivated for textile and seed production. They are a waste product that remains after the fiber is separated from the woody part of the stalk during the retting and decortication processes. For every ton of fiber, approximately 2.5 tons of shives are obtained [1]. In the case of industrial hemp, the woody part from which shives are obtained constitutes 75% of its weight [2,3]. Due to the growing interest in using flax and hemp byproducts, the area under cultivation is increasing systematically. From 2014 to 2024, the total area devoted to fiber flax cultivation in the EU increased from 80,000 to 182,000 hectares [4]. Meanwhile, the area devoted to hemp cultivation increased from 20,540 hectares (ha) in 2015 to 33,020 ha in 2022, and production of the crop rose from 97,130 tons to 179,020 tons [4]. Therefore, it is advisable to find uses for the waste parts of the plant, i.e., the shives. During photosynthesis, hemp and flax absorb carbon dioxide from the atmosphere and convert it into the glucose needed for biomass growth. Consequently, the stems of these plants store carbon from the atmosphere and have a negative carbon footprint. For instance, producing 1 ton of hemp shives requires the plant to absorb approximately 1.8 tons of CO_2_ during its growth [5]. The construction industry is responsible for significant energy consumption, accounting for an estimated 40% of global consumption [6]. This relates to CO_2_ emissions from construction processes, such as cement burning. In 2023, 1.57 billion tons of CO_2_ were emitted globally. To pursue sustainable development in construction and reduce CO_2_ emissions in the coming years, it is advisable to use low-processed materials, such as plant-based materials. These materials not only reduce the carbon footprint of building materials but also have a porous structure that reduces thermal conductivity and, consequently, the energy demand for heating buildings insulated with them.

One example of the use of flax and hemp shives is in lime-binder-based composites, also known as hemp and flax concretes. These materials are primarily used as insulating wall fillers in wooden frames. According to the literature, flax concretes have a bulk density of 515–598 kg/m^3^ [7,8,9,10], while hemp concretes have a wider range of densities, from 330–627 kg/m^3^ [11,12,13,14]. Hemp shives are usually coarser, so hemp concretes have a lower density than flax concretes. The thermal conductivity of hemp concretes made according to selected recipes is approximately 0.075–0.138 W/(m·K) [11,15,16], while that of flax concretes is approximately 0.114–0.170 W/(m·K) [8,17]. Increasing the degree of compaction of these materials significantly increases their thermal conductivity [16,18]. In addition to having good thermal insulation properties, these materials have a high thermal capacity for insulation materials, ranging from 1000 to 1600 J/kg/K [19,20], and they can buffer moisture, which improves thermal and humidity comfort in living spaces [20,21].

Shives are also used to produce insulating boards and panels. An insulating board based on hemp shives bonded with a glue made from crude glycerol and citric acid was developed in Ref. [22]. Boards with densities of 250 and 300 kg/m^3^ were characterized by thermal conductivities of approximately 0.069 and 0.077 W/(m·K), respectively. In another study [23], boards made from hemp shives and Kleiberit urea-formaldehyde resin as a binder were found to have a thermal conductivity of 0.057 W/(m·K) at a density of 300 ± 30 kg/m^3^. Boards based on flax shives bonded with a lignosulfonate binder and epoxy resin were developed in Ref. [24]. These boards had a density of 470–500 kg/m^3^ and a thermal conductivity of 0.074–0.081 W/(m·K).

In addition to their use in organic concrete, shives can be used as loose thermal insulation. Studies [25,26,27] investigated the physical properties of loose flax shives. They showed that, depending on the degree of compaction, the thermal conductivity of shives at a density of 126–159 kg/m^3^ ranged from 0.047 to 0.064 W/(m·K) [25,26]. Furthermore, Ref. [28] demonstrated that particle board made from flax shives using the natural adhesives found in the shives had a thermal conductivity of 0.077 W/(m·K) at a density of 500 kg/m^3^. The physical properties of loose hemp shives were investigated in Refs. [27,29,30,31], among others. The thermal conductivity of hemp shives ranges from 0.049 to 0.058 W/(m·K), with densities ranging from 72 to 124 kg/m^3^ [29,32,33]. In work [27], it was demonstrated that shives are characterized by high total porosity. Hemp shives exhibit a porosity of 78.7%, while flax shives exhibit a porosity of 77.5%. However, they differ in pore size distribution. The average pore diameter is 0.69 µm in HS and 0.39 µm in FS.

High porosity contributes to good thermal insulation properties as well as water and water vapor absorption. In work [25], it was demonstrated that flax shives had a mass water absorption of 195% after 24 h of immersion in water. In turn, hemp shives can reach a mass water absorption of 250% after just 10 min of immersion [34] and up to 400% after 48 h [31]. Research [27] has shown that, in the case of sorptive humidity and an elevated relative humidity (RH) level of 75%, the humidity of hemp shives was 12.9%, while that of flax shives was 13.1%. At the highest RH level (93–100%), the sorptive humidity of hemp shives was 22.8–23.2%, while that of flax shives was 22.7–23.8%. These results demonstrate the materials’ high moisture absorption capacity, especially at elevated RH values. Importantly, at RH = 75%, the humidity reached 12% after just three days, while at RH = 93–100%, the humidity reached 22% after approximately 13 days. Due to their capillary porous structure, which contains perforated pore walls, shives are highly absorbent and can rapidly absorb large amounts of moisture from the air. As indicated in the literature [35], moisture negatively affects the thermal conductivity of fibrous insulating materials. Due to its hydrophobic admixtures and non-porous basalt fiber structure, mineral wool does not absorb as much moisture from water vapor as shives. Research [36] indicates that, even at high relative humidity (RH) levels, the moisture sorption content of mineral wool does not exceed 1%. Similarly, Ref. [37] demonstrated that the mass moisture content of rock wool at 100% RH is 0.9%. Over time, the wool and hydrophobic agents may degrade, increasing the sorption moisture content. While water vapor at temperatures above the dew point may not be detrimental, condensing water vapor within the mineral wool layer of partitions may degrade thermal insulation. Hydrophobic admixtures do not sufficiently protect mineral wool from the effects of water on its thermal conductivity. Research [35] demonstrated that, at a moisture content of 20% by volume, the thermal conductivity coefficient of mineral wool increased to approximately 0.2 W/(m·K), and at 90% moisture content, the coefficient increased to around 0.7 W/(m·K). In the case of cellulose thermal insulation containing capillary pores of macropore size, the impact of moisture on thermal insulation may be smaller despite its high absorption capacity. Water vapor particles could be adsorbed onto the pore walls, leaving air inside the pores. However, this is merely a hypothesis that would require detailed research. A similar research approach was demonstrated in Ref. [38], which investigated adsorption in hemp and flax shives. Certain mechanisms were identified involving water molecules attaching to cell walls composed of biopolymers (cellulose, hemicellulose, and lignin) through hydrogen bonds formed by their hydroxyl groups. Despite the larger pore sizes and greater porosity in hemp shives, the moisture content in flax and hemp shives was similar. Therefore, it was concluded that the large pores in the shives do not affect adsorption; the main factor is the availability of hydroxyl groups. Nevertheless, the large pores in the shives may affect thermal conductivity, regardless of the material’s adsorption capacity. Therefore, it is worthwhile to investigate the effect of moisture on the thermal conductivity of flax and hemp shives, as this can significantly impact the thermal performance of insulated elements under variable humidity conditions. The reduced effect of moisture on the thermal conductivity of shives compared to mineral wool may improve living comfort and reduce heat loss in buildings.

In recent years, plant-based insulation materials have been the subject of extensive research, with particular attention given to hemp–lime composites and other highly porous plant-derived materials. Numerous studies have investigated their thermal performance, moisture buffering capacity, and thermo-hygrometric behavior at both material and building scales, including experimental investigations of hemp–lime plasters, composite insulation materials, and full-scale buildings under different climatic conditions [39,40,41]. These works indicate that heat and moisture transport processes in hemp-based materials are strongly coupled and significantly influenced by material composition and pore structure.

Comparative investigations of hemp and flax materials have further demonstrated that fibers and shives exhibit distinct moisture and heat sorption behavior, highlighting the complexity of hygrothermal phenomena in plant-based insulation materials [38]. In addition, studies on hemp–lime composites have shown that moisture fixation and buffering play a crucial role in shaping their thermal and hygric performance [18].

Despite this growing body of research on hemp-based materials, experimental data on loosefill flax and hemp shives, particularly under controlled moisture conditions and varying bulk density, remain limited. This gap motivates the experimental and numerical investigations presented in this study.

This study examined the thermal conductivity of locally sourced flax and hemp shives with varying moisture levels. It is a continuation and extension of previous research [25,27,29]. Based on the results, a simulation was performed using Delphin 6.1.5 software to model a wall partition insulated with shives at different moisture levels.

## 2. Materials and Methods

### 2.1. Materials

**Figure 1:**
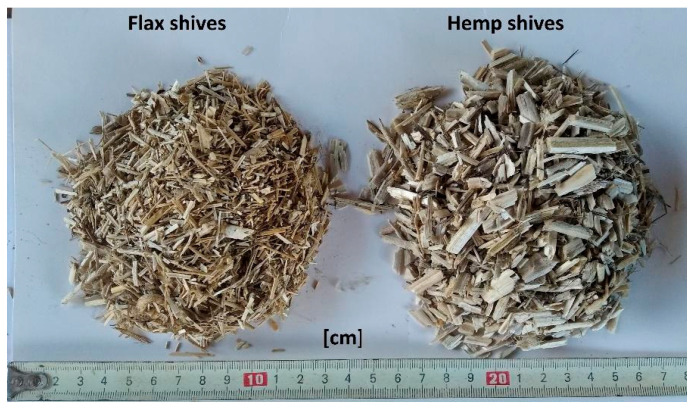
Flax (**left**) and hemp (**right**) shives used in the presented research.

For this study, flax shives (FS) of the Polish variety Modran and hemp shives of the Polish variety Białobrzeskie were selected. Flax shives were produced by the Institute of Natural Fibers and Medicinal Plants (Poznań, Poland), while hemp shives (HS) were produced by the Polish producer Podlaskie Konopie (Białystok, Poland). HS were high-quality intended for use in construction, mainly as a filler in a lime-hemp composite, also in our earlier works [13,14]. They were characterized by a low dust and fiber content. The moisture content as delivered from the manufacturer was 9%, while porosity measured using MIP (mercury intrusion porosimetry) was 78.7% [27]. The measurements were conducted with the use of AutoPore IV 9500 (Micromeritics Instrument Corporation, Nocross, GA, USA). The flax shives’ moisture content in the air-dry state was about 8.8%, while porosity by MIP was 77.5% [27]. The FS mixture was used in previous research as fillers in a lightweight lime-based composite [42,43] and as a loose insulating material [25]. Both hemp and flax shives were obtained from straw subjected to retting in the field. The shives used in the research are presented in Figure 1. The detailed properties of both shives are presented in [25,27,29].

### 2.2. Methods

#### Thermal Conductivity

The thermal conductivity measurements were carried out with the Laser Comp FOX 602 apparatus using the heat flux sensor method based on the EN 12667 standard [44]. The measurements of the thermal conductivity coefficient were made at the average temperature of 10 °C, with 0 °C on the bottom plate and 20 °C on the top plate. The mean temperature of 10 °C corresponds to the typical conditions of the climate of Central Europe [45,46,47]. The measurements were conducted automatically using the WinTherm32 software. The thermal conductivity coefficient is defined by the Fourier law (1) based on one-dimensional heat flow:

(1) $$q=-\lambda \frac{dT}{dx},$$

where *q* is the heat flux density flowing through the sample (W/m^2^), *λ* is the thermal conductivity coefficient of the sample (W/(m·K)), and *dT*/*dx* is the temperature gradient on the isothermal flat surface of the sample (K/m).

**Figure 2:**
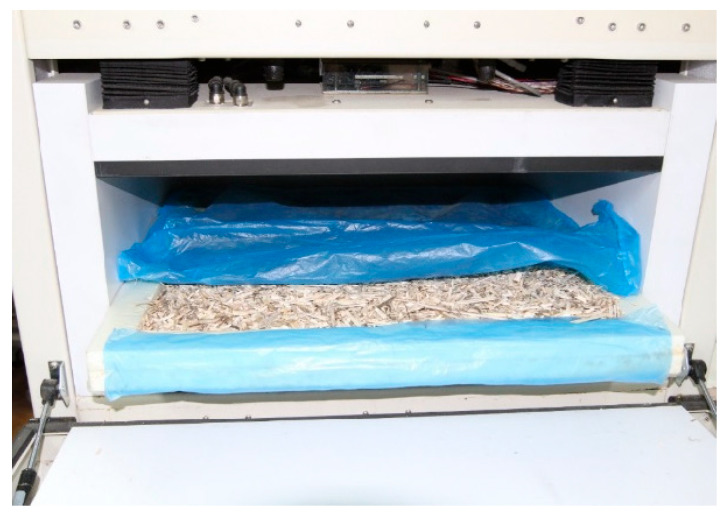
A prepared sample of dry HS placed in the HFM apparatus [29].

Figure 2 presents the sample places in an HFM apparatus. The tests were performed for dry and sorption-moistened samples. Samples preparation is described in the separate paragraph.

### 2.3. Sample Preparation

The shives were randomly selected from bulk packages. This allowed us to obtain a wide variety of tested materials, which correspond to their natural characteristics. The manufacturers typically package shives randomly. This means that some packages contain mostly large or small particles. Based on our experience, we randomly sampled shives from packages [25,27,29] to ensure consistent particle size. The tests were performed on samples made from dry and moistened shives. The sample preparation methods varied depending on the degree of wetting or drying of the selected material.

The flax and hemp shives were divided into three parts. The preparation time for each part varied depending on the desired final form. The first part was tested as a dry material. The shives were placed in flat trays and placed in a laboratory dryer at 105 °C until a constant mass was achieved.

The second part of the shives was subjected to moisture absorption at 80% relative humidity and 30 °C temperature. The intention was to achieve the highest possible moisture content of the samples. For this purpose, the previously dried shives were placed in a climate chamber on flat trays for seven days. During the wetting process, the shives were frequently mixed up to ensure the properties were as uniform as possible throughout the material. The 7-day period was determined based on previous experience [27]. A maximum relative humidity (RH) level of 80% was assumed to be critical due to the risk of surface condensation on the wall and mold growth.

The third part of the shives was subjected to moisture sorption under natural laboratory conditions, i.e., temperature of 20–23 °C and relative humidity ranging from 35–55%. The shives were placed in flat containers and periodically mixed. Conditioning lasted for four months to achieve constant mass.

Moisture content of the tested samples was determined gravimetrically using a Wamed SUP 100W (Wamed, Warszawa, Poland) laboratory drying oven and a Radwag AS 310X (Radwag, Radom, Poland) precision scale. Moisture content tests were performed on 5 g wet samples. Moisture contents of the tested samples are presented in Table 1.

**Figure 3:**
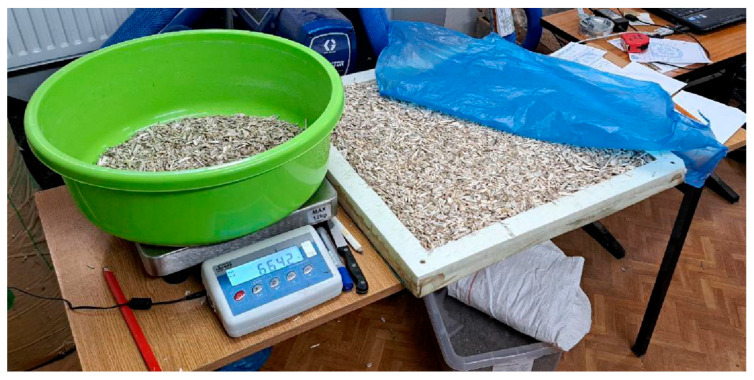
Dry hemp shives sample preparation.

The process of forming samples from the prepared material was uniform (Figure 3). The shives were placed in a frame made of extruded polystyrene with dimensions: 600 × 600 mm^2^ (external), and 520 × 520 mm^2^ (internal), as well as a height of 45 mm. To prevent the condensation of water vapor, the frame with the test sample was covered with a thin foil. Initially, the material was spread evenly by hand within the frame to ensure it lay flat. To increase the density of the mixture, shives were added and spread by hand before being loaded with a weighting element of known mass. Nevertheless, due to the hardness of shives, it was not easy to achieve the assumed density of material. The loading itself was ineffective; it was necessary to manually adjust the shive mixture to ensure that the shives of different sizes fit together properly and fill the frame completely. The specific nature of these natural, lightweight, loose, and unsorted materials makes it difficult to maintain the density parameter with strict repeatability. Densities of the tested samples are presented in Table 1. The density given for wet shives refers to the dry material. The density range for the dry material tested is wider than for wet shives.

For each of the 9 prepared dry samples of each shive kind, 12–13 results of the thermal conductivity coefficient were obtained. Steady-state conditions were maintained throughout the tests. Each sample was thermally stabilized in the FOX 602 measuring chamber (TA Instruments, New Castle, DE, USA) before the instrument started the thermal conductivity determination. During subsequent measurements, the steady-state temperature on both plates was maintained. For each of the 4 prepared moistened samples of each shive kind, 15 results of thermal conductivity were obtained. With each measurement repetition, the thermal conductivity value of wet samples decreases as moisture migrates toward the colder plate. Therefore, after many repetitions, the sample exhibits an uneven moisture distribution. Based on previous studies with moistened mineral wool, wood wool, and cellulose fibers, we repeated the reading more often [48]. Only those readings in which thermal conductivity does not drop dramatically are accepted for analysis. Thus, only the first 10 measurements performed for each sample were selected for further analysis [48].

## 3. Results

### 3.1. Thermal Conductivity

**Figure 4:**
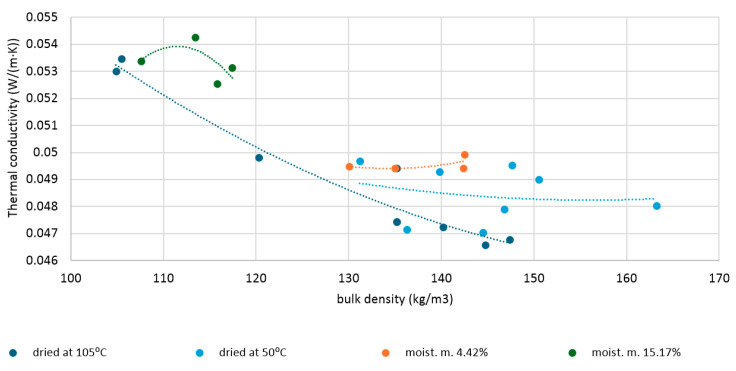
Thermal conductivity of dry and moistened flax shives, depending on bulk density.

Figure 4 shows the average thermal conductivity values of dry and moistened flax shives samples. The thermal conductivity of dry shives depends on their density. Within the analyzed density range (104–147 kg/m^3^), the thermal conductivity tended to decrease. The highest thermal conductivity (λ) achieved was 0.0534 W/(mK), and the lowest was 0.0466 W/(mK). For comparison, the thermal conductivity coefficient of dry hemp shives with a density of 110 kg/m^3^ was found to be 0.048 W/(m·K) in the work [49], while that of shives with a density of 155 kg/m^3^ was found to be 0.058 W/(m·K). According to research [26], flax shives with a density of 126–154 kg/m^3^ were characterized by a thermal conductivity of 0.057–0.064 W/(m·K). Shives with a moisture content of 4.42% had values ranging from 0.0494 to 0.0500 W/(mK). The obtained thermal conductivity results exhibit a parabolic distribution, with the lowest value obtained for the sample with the middle density. Shives with a moisture content of 15.17% had thermal conductivities ranging from 0.0525 to 0.0543 W/(mK). In this case, the results were random; the sample with the middle density had the highest thermal conductivity.

Based on the analysis of thermal conductivity versus bulk density curves for loose, fibrous materials [50,51,52], it can be assumed that the lowest thermal conductivity for dry flax shives would be obtained at a density no lower than the second-to-last density tested (144.72 kg/m^3^). A parabolic distribution curve with a coefficient of determination of 0.943 was proposed to approximate the distribution of thermal conductivity versus density. This allowed for a detailed analysis of the effect of moisture on the thermal conductivity of flax shives. For shives’ densities above 120 kg/m^3^, increasing the density of dry shives significantly reduced the thermal conductivity coefficient. In contrast, for shives with a moisture content of 4.42% caused, stabilization or even a slight increase in thermal conductivity occurred with increasing density above 130 kg/m^3^. This is due to the increased water content per unit volume of shive and the reduction in air volume.

It is worth mentioning that the results presented in this work differ slightly from the previous investigation described in [25]. The thermal conductivity results presented here are slightly lower due to more thorough drying of the shives. In [25], flax shives were dried at 50 °C, while in the current study, at 105 °C. As demonstrated in [38,53], the moisture present in shives may largely exist as water bound by hydrogen bonds to hydroxyl groups. Therefore, a temperature of 50 °C may not have been sufficient to completely remove this bound water. However, this is only a hypothesis and requires further investigation. In the study [54], wood was dried at both 40 °C and 80 °C. This resulted in the same moisture content, but a longer drying time was required at 40 °C. The effect of drying at 105 °C was not tested in this study.

**Figure 5:**
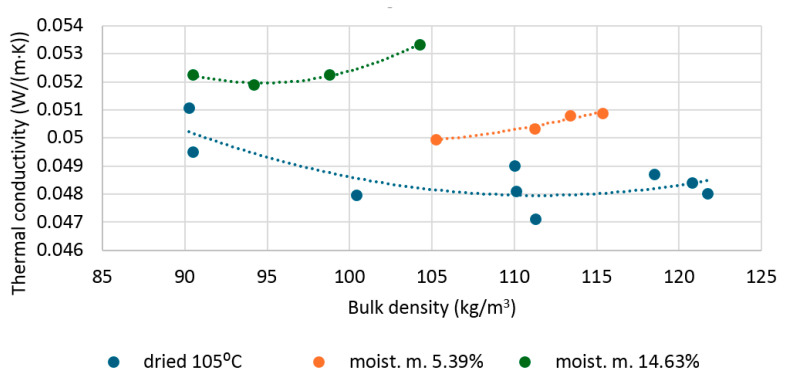
Thermal conductivity of dry and moistened hemp shives, depending on bulk density.

Figure 5 shows the average values from thermal conductivity measurements of dry and moistened hemp shive samples. The thermal conductivity of dry shives depends on density. Within the analyzed density range of 90 to 121 kg/m^3^, it decreased until reaching a density of approximately 110 kg/m^3^ and then increased. The highest thermal conductivity (λ) achieved was 0.0511 (0.0495) W/(m·K), and the lowest was 0.0471 W/(m·K). The distribution of results is parabolic. For shives with a moisture content of 5.39%, an increasing trend in thermal conductivity is visible as density increases within the analyzed range. The lowest measured thermal conductivity was 0.0499 W/(mK), and the highest was 0.0509 W/(mK). For shives with a moisture content of 14.36%, a parabolic distribution of measured thermal conductivities is visible. The lowest measured thermal conductivity, 0.0519 W/(mK), was for a sample with a density of 94.18 kg/m^3^, while the highest, 0.0533 W/(mK), was for a density of 104.23 kg/m^3^.

The parabolic distribution of the presented thermal conductivity is strongly related to the compaction of the tested material. As the density of the loose material increases, the pores close, reducing the convective component and increasing the air thermal conduction. Further increases in material density cause excessive volume expansion of the solids, i.e., the fibers, thus the solid conduction increases while the air volume decreases, meaning that the trapped air becomes less and less of an insulator.

### 3.2. The Influence of Moisture on the Thermal Conductivity of Shives

Moisture plays a significant role in the heat transfer process of porous materials. As a material’s moisture content increases, air escapes from its pores, which then fills with water. For most building materials, this results in a substantial increase in thermal conductivity with just a few percent of mass moisture content. The manner in which moisture affects thermal conductivity varies by material. For cellular concrete, for example, the relationship is linear [55,56]. For wood fiber insulation and rock wool, the function is linear for materials with low thermal conductivity; however, curves are observed for materials with higher conductivity [57,58]. Similarly, as shown in Figure 4, in the case of hemp shives with a moisture content of 5.39%, thermal conductivity increases with increasing density, but at lower densities (above 105 kg/m^3^). In contrast to flax shives, at higher moisture values (approximately 15%), the relationship between thermal conductivity and material density is typically parabolic, as in the case of dry shives, but the extreme (minimum λ value) was reached at a significantly lower density level.

In the case of materials wetted by sorption, many thermal conductivity increase functions can be approximated linearly. Budaiwi et al. [59] investigated the thermal conductivity of fiberglass under different moisture levels and found a linear relation between them. For natural materials, linear relationships may be limited to a smaller moisture range than for most building materials, such as ceramics or EPS. The authors see a need for a detailed analysis of whether the linear adjustment of the effect of humidity on the thermal conductivity of thermal insulation made of shives does not lead to their overestimation. Therefore, experiments are required to determine the thermal properties of materials with low thermal conductivity of organic origin under variable humidity conditions. Although such studies have been conducted for many materials, including the aforementioned wood fibers, no results are available for flax and hemp shives. There is also a gap in numerical modeling of the hygrothermal performance of natural-based materials subjected to moisture. Manufacturers WUFI and Delphin point to the need to study the properties of wetted organic materials [60,61].

Considering that the thermal conductivity of shives depends on moisture content and is not linear, the authors compared the values determined from the linear function given by Equation (2) with the values obtained experimentally.

(2) $$\lambda_{2}=\lambda_{1}+\Psi \cdot \lambda_{w,T}$$

where *λ*_1_—is the thermal conductivity coefficient of dry material (W/(m·K)), *λ*_2_—is the thermal conductivity coefficient of a moistened material (W/(m·K)), *Ψ*—volumetric moisture content (m^3^/m^3^), *λ_w,T_*—is the thermal conductivity coefficient of water in a specific temperature (W/(m·K)).

The linear model is generally used for porous materials and, for simplicity, has also been adopted for shives. However, certain limitations should be noted when applying it to porous cellulose materials. It does not account for the specific pore arrangement in shives (a network of parallel channels connected by perforations in the cell walls), which may be important for moisture adsorption and, consequently, for the material’s thermal conductivity. The model also does not account for the heat capacity of these materials, which determines heat buffering, or for thermal phenomena during phase transitions. However, in practice, according to applicable Polish regulations (Technical Conditions), the selection of wall thickness and layer arrangement depends solely on thermal conductivity, not heat capacity.

Graphs 6–9 show a comparison of thermal conductivity values determined using Equation (1) and measured values.

**Flax shives**

**Figure 6:**
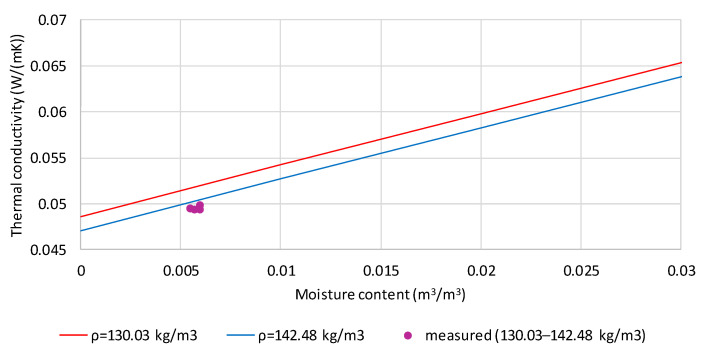
Comparison of measured and calculated values of thermal conductivity of flax shives moistened to the mass content of 4.42% and densities in the range 130.03 to 142.48 kg/m^3^.

The lines in Figure 6 correspond to the calculated thermal conductivity values for the samples’ extreme densities (130.03 and 142.48 kg/m^3^), and the purple points indicate the thermal conductivity of flax shives with 4.42% moisture by mass. The moisture volume fraction for the four tested samples is between 0.005461 and 0.005984 m^3^/m^3^. The measured values are slightly lower than the calculated values (0.00053–0.00218 W/(m·K)), corresponding to 1.1–4.4% of the measured values. This difference decreases with increasing density.

**Figure 7:**
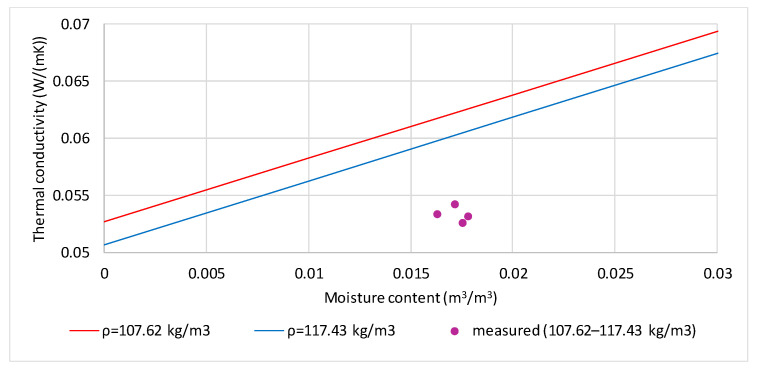
Comparison of measured and calculated values of thermal conductivity of flax shives moistened to the mass content of 15.17% and densities in the range 107.62 to 117.43 kg/m^3^.

Figure 7 shows a comparison of the measured and calculated thermal conductivity values of flax shives with 15.17% moisture by mass (purple points). The lines correspond to the calculated thermal conductivity values for the samples’ extreme densities (107.62 and 117.43 kg/m^3^). The volume fraction of moisture for the four tested samples ranges from 0.016326 to 0.017814 m^3^/m^3^. The difference between the measured and calculated thermal conductivity values is significant (0.00679–0.00897 W/(m·K)), corresponding to 12.5–16.3% of the measured values.

For comparison, the study [26] tested hemp shives with a density of 130 kg/m^3^ and a moisture content of 10–25%, corresponding to an RH of 50–95%. The thermal conductivity was found to be between 0.057 and 0.075 W/(m·K).

**Hemp shives**

**Figure 8:**
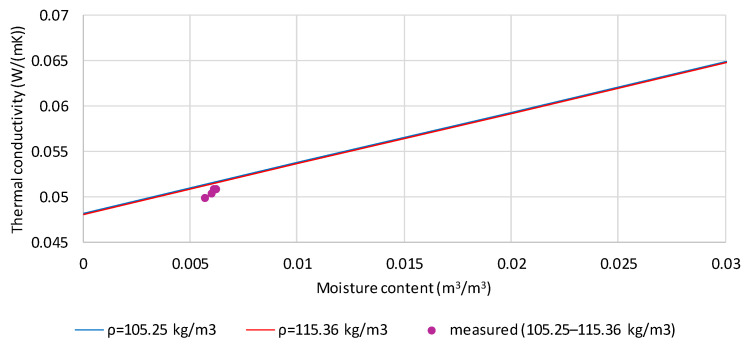
Comparison of measured and calculated values of thermal conductivity of hemp shives moistened to the mass content of 5.39% and densities in the range 105.25 to 115.36 kg/m^3^.

Figure 8 shows a comparison of the measured and calculated thermal conductivity values of hemp shives with a moisture content of 5.39% (purple points). The lines correspond to the calculated thermal conductivity values for the samples’ extreme densities (105.25 and 115.36 kg/m^3^). The volume fraction of moisture for the four tested samples ranges from 0.005668 to 0.006213 m^3^/m^3^. The difference between the measured and calculated values is small (0.00057 to 0.00137 W/(m·K)), corresponding to 1.1–2.7% of the measured values.

**Figure 9:**
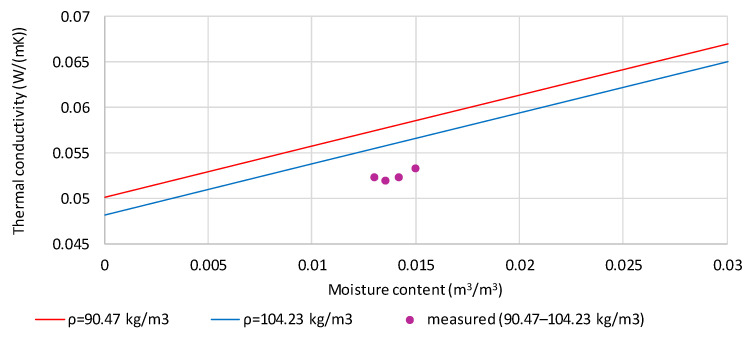
Comparison of measured and calculated values of thermal conductivity of hemp shives moistened to the mass content of 14.36% and densities in the range 90.47 to 104.23 kg/m^3^.

Figure 9 presents a comparison of measured and calculated values of thermal conductivity of hemp shives moistened to the mass content of 14.36% (purple points). The lines correspond to the calculated thermal conductivity values for the samples’ extreme densities (90.47 and 104.23 kg/m^3^). The volume fraction of moisture for the four tested samples ranges from 0.012992 to 0.014968 m^3^/m^3^. There is a significant difference (0.00441 to 0.00605 W/(m·K)) between the measured and calculated thermal conductivity values, corresponding to 8.4–11.3% of the measured values. The higher the density of the tested samples, the greater the difference.

For comparison, work [49] showed that increasing the volumetric moisture content of randomly oriented hemp shives (110 kg/m^3^) from 0% to 20% increased thermal conductivity from 0.048 W/(mK) to about 0.09 W/(mK). In another study [62], thermal insulation based on hemp shives with a density of approximately 200 kg/m^3^ was tested. It was shown that, at a mass moisture content of around 10%, there was a negligible increase in thermal conductivity compared to a dry sample. However, at a mass moisture content of 85%, the thermal conductivity increased approximately twice.

## 4. Hygrothermal Simulations

The simulation aimed to determine the thermal and moisture state of frame walls insulated with flax and hemp shives. The simulation used laboratory-measured material characteristics, such as sorption isotherms [37] and the thermal conductivity of dry and wet shives. Delphin 6.1.5, which the authors used, includes the temperature influence on thermal conductivity. Kosiński and Patyna demonstrated that the relative humidity of loose insulation in frame walls can reach 99% at the cold edge [63]. The following calculations aim to relate shive moisture to heat losses.

**Figure 10:**
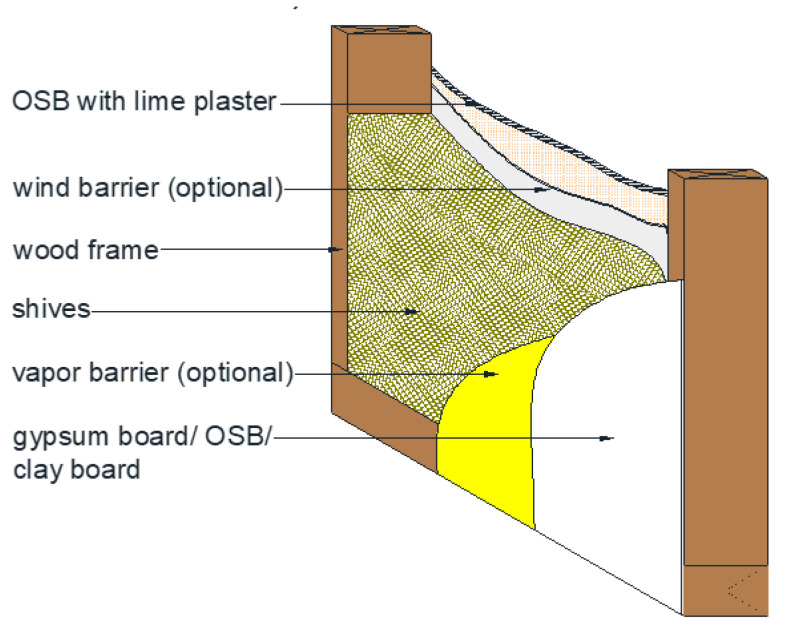
Schematic model of a frame wall insulated with shives.

Simulations were performed on models of wooden frame walls filled with loose hemp and flax shives. The exterior of the walls was covered with OSB boards and lime plaster, and the interior was covered with OSB boards, gypsum boards, and clay boards. According to Polish regulations [64], a thermal insulation material thickness of 26 cm with 70 cm spacing between wooden studs allows the frame wall to achieve a heat transfer coefficient of 0.20 W/(m^2^K). Figure 10 shows a cross-section example of a frame wall filled with loose shives.

Simulations were performed for models with and without a vapor barrier to assess its impact on the moisture content of the models. No wind barrier was used.

Table 2 contains the basic material data used for the thermal and humidity simulations. Additionally, a vapor barrier foil with an S_d_ value of 100 m was used.

**Figure 11:**
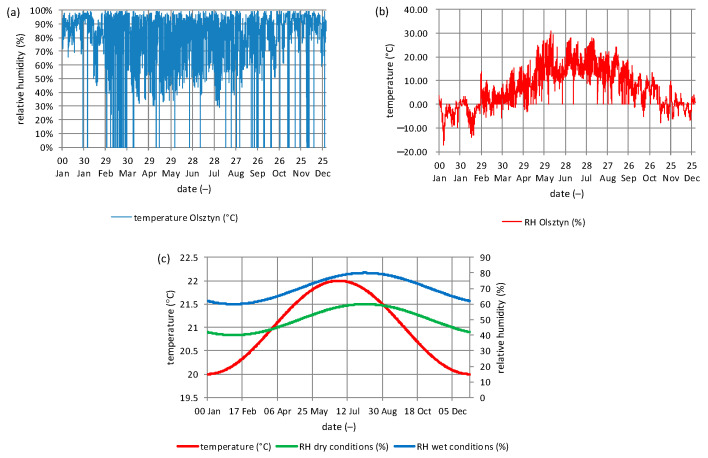
Climate conditions and model for simulation: (**a**) relative humidity and (**b**) temperature for Olsztyn, based on Typical Meteorological Year (**c**) internal temperature and relative humidity [63].

The external boundary conditions were based on the typical meteorological year in Olsztyn [65]. The internal conditions were set as a sinusoidal temperature and relative humidity. The temperature was set to range from 20 to 22 °C, peaking at the turn of June and July. The relative humidity was set to two variants: dry, with a minimum of 40% at the turn of February and March, peaking at 60% in September; and humid, with a minimum of 60% at the turn of February and March, peaking at 80% in September. The distribution of temperature and relative humidity is shown in Figure 11a–c.

**Hemp shives**

**Figure 12:**
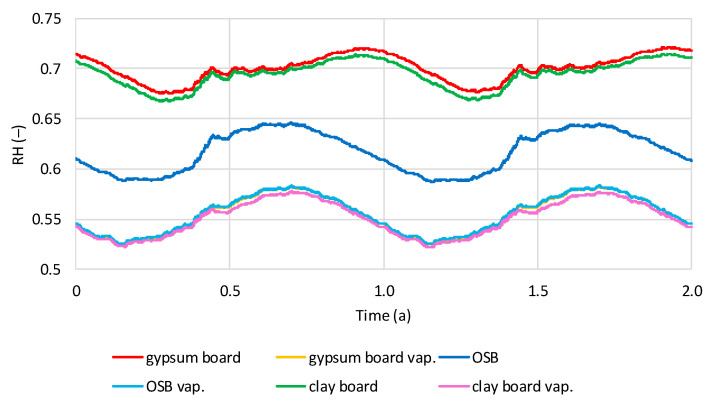
Calculated relative humidity in hemp shives in tested models with and without vapor barrier, regarding the time in dry internal conditions.

The values presented below are the results of calculations for thermodynamically stable models over a two-year period. The stabilization time was two to four years. Figure 12 shows the average relative humidity (RH) values in the hemp shive insulation layer for two wall variants, one with a vapor barrier and one without, under dry interior humidity conditions. The influence of the vapor barrier and internal wall cladding is clearly visible. A sinusoidal variation in RH over time is visible in all cases. Models with OSB boards exhibit the highest RH in August–September and the lowest in March–April. The same trend is visible for models with gypsum and clay boards and a vapor barrier. Models with gypsum and clay boards without a vapor barrier have the highest RH in November–December and the lowest in May–June. The lowest humidity is found in models without a vapor barrier and with OSB cladding (0.59–0.65), while for gypsum and clay boards, the humidity is comparable (0.67–0.72). In all models, RH is similar (0.52–0.58).

**Figure 13:**
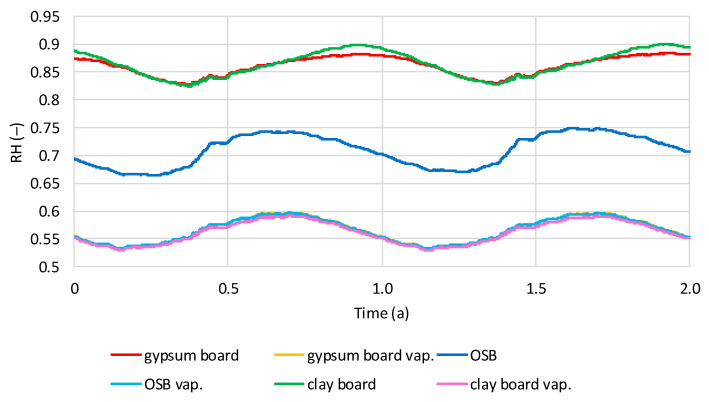
Calculated relative humidity in hemp shives in tested models with and without vapor barrier, regarding the time in wet internal conditions.

Figure 13 shows the average relative humidity (RH) values in the hemp shive insulation layer for two wall variants, one with a vapor barrier and one without, under humid interior conditions. Similar to dry conditions, sinusoidal RH variations can be observed in the shives. Models with a vapor barrier and models with OSB boards without a vapor barrier reach the lowest humidity in February–March and the highest humidity in August–September. Models without a vapor barrier and with gypsum or clay boards reach the highest RH in November–December and the lowest in May–June. In humid conditions, the lowest RH occurs in the OSB-facing model (0.66–0.75), intermediate in the gypsum board model (0.82–0.88), and highest in the clay board model (0.82–0.90). Using a vapor barrier significantly reduces RH in the thermal insulation layer. In all models with a vapor barrier, RH in the shive layer ranges from 0.53 to 0.59, depending on the season.

**Figure 14:**
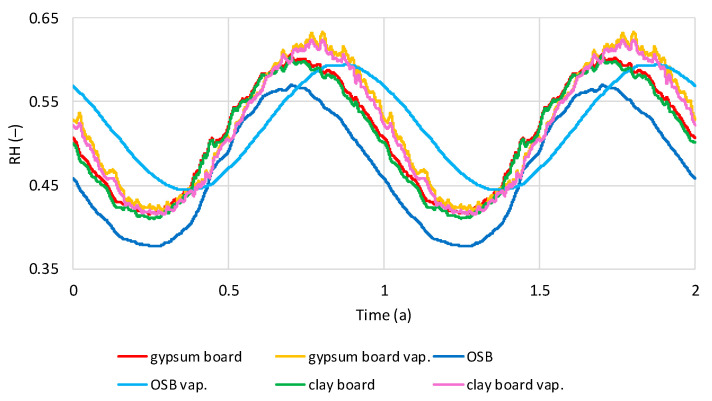
Calculated relative humidity in interior sheathing boards in tested models filled with hemp shives with and without vapor barrier, regarding the time, in dry internal conditions.

**Figure 15:**
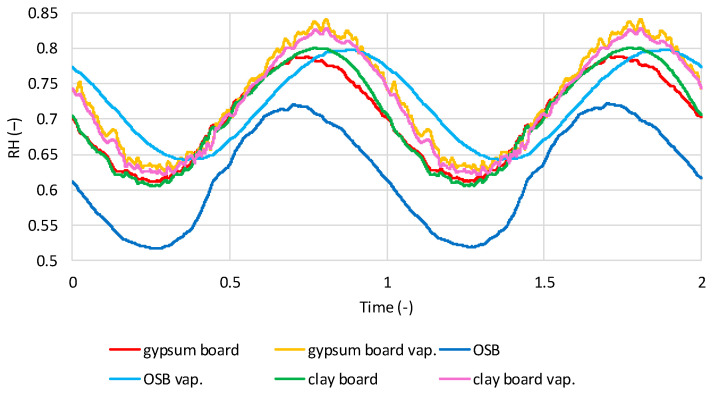
Calculated relative humidity in interior sheathing boards in tested models filled with hemp shives with and without vapor barrier regarding the time, in wet internal conditions.

The presented relative humidity results from thermal insulation correspond to the RH distribution in the inner sheathing boards (Figure 14 and Figure 15). Figure 14 presents the RH distribution in the boards of the models with and without a vapor barrier under dry internal conditions. In all analyzed cases, a sinusoidal trend depending on the season is visible. The boards of the models without a vapor barrier gain and lose humidity in similar cycles, reaching the lowest RH in March–April and the highest in September. In the models with a vapor barrier, OSB boards reach the lowest humidity in May and the highest in November, while gypsum and clay boards reach the lowest in March–April and the highest in October. In the models without a vapor barrier, OSB boards are characterized by the lowest RH (0.38–0.57), while in gypsum and clay boards, the RH oscillates in the range of 0.41–0.61. In models with a vapor barrier, RH is higher, for OSB 0.44–0.59, for gypsum and clay boards in the range of 0.42–0.63.

Figure 15 shows the relative humidity (RH) distribution in models with and without a vapor barrier under humid indoor conditions. Similar to Figure 13, a sinusoidal trend in RH changes is visible. For models without a vapor barrier, the lowest RH occurs in March–April and the highest in September for OSB and gypsum boards, and in November for clay boards. In models with a vapor barrier, gypsum and clay boards reach the lowest RH in March–April and the highest in October–November. OSB boards reach the lowest RH in May and the highest in November–December. Without a vapor barrier, gypsum and clay boards reach 0.61–0.80, and OSB reaches 0.52–0.72. In models with a vapor barrier, gypsum and clay boards reach 0.62–0.84, with slightly lower RH for clay boards. OSB reaches 0.64–0.80.

**Flax Shives**

**Figure 16:**
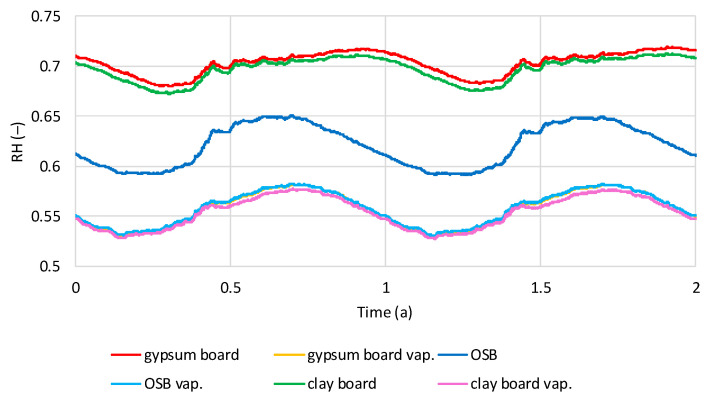
Calculated relative humidity in flax shives in tested models with and without vapor barrier, regarding the time in dry internal conditions.

Figure 16 shows the average relative humidity (RH) values in the flax shive insulation layer for two wall variants: one with a vapor barrier and one without. The conditions are dry interior humidity. The influence of the vapor barrier and interior wall cladding is clearly visible. All cases exhibit a sinusoidal variation in RH over time. Models with OSB boards, with or without a vapor barrier, reach the lowest RH in March–April and the highest in August–September. Models without a vapor barrier and with gypsum and clay boards reach the lowest RH in May and the highest in November. Models with a vapor barrier and gypsum and clay boards reach the lowest RH in February–March and the highest in August–September. The lowest RH in models without a vapor barrier is for OSB boards (0.59–0.65), and the highest is for gypsum and clay boards (0.68–0.72). For models with a vapor barrier for all boards, the range is 0.53–0.58.

**Figure 17:**
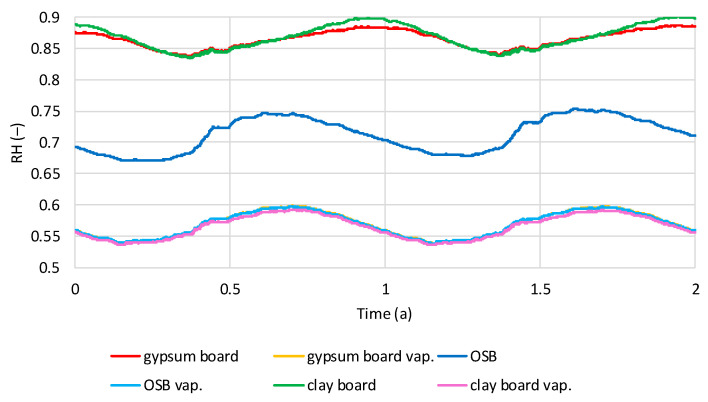
Calculated relative humidity in flax shives in tested models with and without vapor barrier, regarding the time in wet internal conditions.

Figure 17 shows the average RH values in the insulation layer made of flax shives for two wall variants: with and without a vapor barrier, for damp internal humidity conditions. As with dry conditions, a sinusoidal RH variation can be observed in the shives. Models with a vapor barrier achieve the lowest humidity in February–March, and the highest in August/September. Models without a vapor barrier, with gypsum and clay boards, achieve the highest RH in December–January, the lowest in May–June. OSB is at its lowest in March–April and at its highest in August–September. For models without a vapor barrier, the RH for OSB is 0.67–0.75, for gypsum boards 0.84–0.89, and for clay boards 0.83–0.90. In models with a vapor barrier, the RH for all boards is 0.54–0.60.

**Figure 18:**
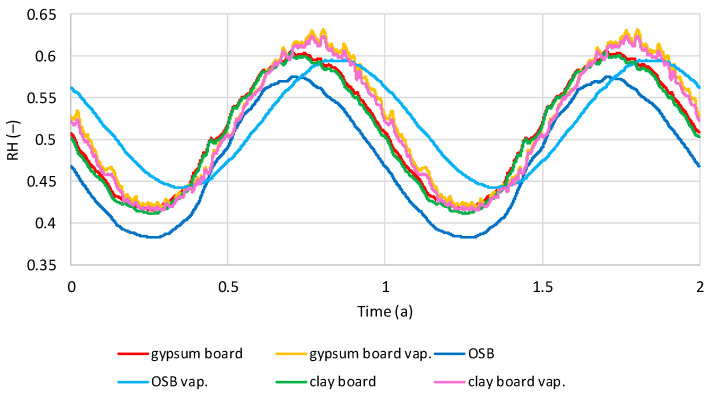
Calculated relative humidity in interior sheathing boards in tested models filled with flax shives with and without vapor barrier, regarding the time, in dry internal conditions.

Figure 18 illustrates the changes in relative humidity (RH) within the inner sheathing boards of models filled with flax shives under dry interior conditions. In all analyzed cases, a seasonal sinusoidal trend is visible. Boards in models without a vapor barrier exhibit similar cycles of moisture gain and loss, reaching the lowest RH in March–April and the highest in August–September. In models with a vapor barrier, OSB boards reach the lowest RH in May and the highest in November. Gypsum and clay boards reach the lowest RH in March or April and the highest in October. Without a vapor barrier, OSB boards have the lowest RH (0.38–0.58), while gypsum and clay boards have an RH range of 0.41–0.61. In models with a vapor barrier, RH is higher: 0.44–0.60 for OSB and 0.42–0.63 for gypsum and clay boards.

**Figure 19:**
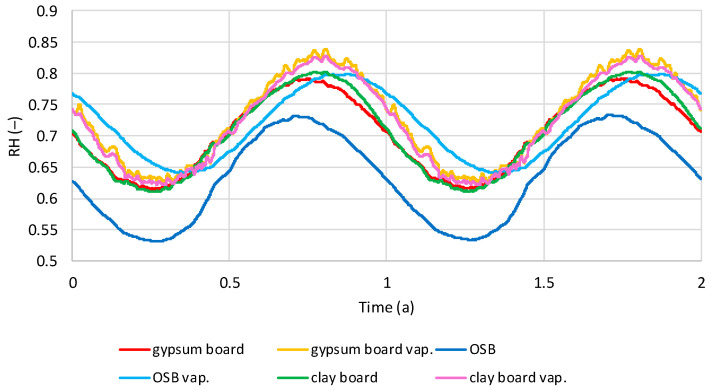
Calculated relative humidity in interior sheathing boards in tested models filled with flax shives with and without vapor barrier, regarding the time, in wet internal conditions.

Figure 19 shows the changes in RH in the inner sheathing boards in models filled with flax shives for internal humid conditions. In all analyzed cases, a sinusoidal trend dependent on the season is visible. Boards in models without a vapor barrier gain and lose moisture in similar cycles, reaching the lowest RH in March–April and the highest in August–September. In models with a vapor barrier, OSB boards reach the lowest humidity in May and the highest in November, while gypsum and clay boards reach the lowest in March–April and the highest in October. In models without a vapor barrier, OSB boards are characterized by the lowest RH (0.53–0.73), while in gypsum and clay boards, the RH oscillates in the range of 0.61–0.80. In models with a vapor barrier, RH is higher, for OSB 0.64–0.80, for gypsum and clay boards in the range of 0.62–0.84.

Without a vapor barrier, the relative humidity (RH) of the thermal insulation can rise to high levels: up to 72% in dry interior conditions and up to 90% in humid conditions. RH levels are slightly lower when OSB boards are used, reaching up to 65% and 75%. OSB boards have higher diffusion resistance, so less moisture reaches the shives.

An occurrence of vapor barrier (s_d_ at least 100 m) results in lower relative humidity of the thermal insulation, up to 58% in dry conditions and up to 60% in humid conditions. No significant influence of the sheathing board on the RH of the thermal insulation was found in this case.

According to research described in [27], mold grew in desiccators where shives were stored at RHs of 93% and 100%. Therefore, there is a high risk of mold growth in the shives with internal sheathing boards made of clay or gypsum board. Soaking the shives in an alkaline solution can protect them from biological corrosion. In one study [66], hemp fibers were treated with a solution of NaOH and Ca(OH)_2_. However, this would involve the absorption of large amounts of water by the shives and the need to dry them. However, in future analyses of the external wall layer system, it would be reasonable to consider the possibility of surface and intra-layer condensation in order to assess the possibility of biological corrosion developing on a wall containing organic insulation.

## 5. Limitations, Challenges, and Future Perspectives

The authors are aware of the limitations of the research. Sample shaping was difficult. A significant amount of material must be moistened and then analyzed before any conclusions can be drawn. The research described in this article focused on two varieties of flax and hemp shives. Other varieties may have different moisture absorption capacities and, therefore, different thermal conductivity. Furthermore, thermal conductivity measurements of moist samples are always associated with thermal diffusion, which influences the interpretation of results.

The simulation presented in the article is of a demonstration character. The authors tested material properties, while the simulation is concerned with the wall elements. The results of the simulation gave us insights into the cladding boards and vapor barrier’s impact on the hygrothermal state of the analyzed walls. From our point of view, these are practical results of the simulations.

The material and numerical tests we conducted have brought us closer to the next step: building and testing macro-scale models. We plan to construct walls filled with shives and study their thermal and moisture parameters in natural conditions.

## 6. Conclusions

Analyses confirm that shives, even in dry indoor conditions, are almost constantly exposed to relative air humidity of 50% or higher. Due to their hygroscopicity, this affects the material’s actual moisture content, meaning that the thermal conductivity of a completely dry material should not be used in heat loss calculations under operational conditions. Therefore, in the moderate Central European climate, it is recommended to condition samples for thermal conductivity testing at 50% RH, as this best reflects the behavior of shives in building envelopes.

The test results indicate that samples conditioned under laboratory conditions exhibit moisture levels most similar to those observed in real structures. The linear simplification of the effect of moisture on thermal conductivity used in the literature (Equation (1)) leads to an overestimation of the λ value, especially in samples with higher moisture content. At 4–6% moisture content, the overestimation is only 1–4%, allowing the linear model to be used approximately. At higher moisture contents, the error increases to 8–16%, significantly limiting its usefulness.

A sinusoidal annual pattern of moisture changes was observed in both the shives and the sheathing boards. The type of sheathing board used has a significant impact on moisture levels in the thermal insulation. Models with OSB boards exhibit lower moisture content in the shives due to the higher diffusion resistance of wood-based boards. In systems with OSB or a vapor barrier, the lowest RH values in the shives are generally recorded in February–March and the highest in August–September. In models with boards with lower diffusion resistance, such as gypsum boards or clay boards, without a vapor barrier, humidity minima occur in May–June and maxima in November–December.

The presence of a vapor barrier limits moisture in the thermal insulation layer while simultaneously increasing the humidity of the sheathing panels. Simulations also showed that both flax and hemp achieve similar RH levels inside the partition, indicating that the type of sheathing panel and the presence of a vapor barrier are more important in shaping humidity conditions than the choice of shive species.

Our results demonstrate that the choice of sheathing and vapor barrier boards is more important than the choice of shive species for controlling humidity in the walls.

Studies of moistened shives and numerical simulations have provided insight into the moisture distribution in frame walls filled with shives. The next step will be research on real models placed in a Hot Box climatic chamber.

## Figures and Tables

**Table 1:** Moisture content and density ranges of the tested shive samples.

Moisture Content (%)	Flax Shives Densities (kg/m^3^)	Hemp Shives Densities (kg/m^3^)
dry		
0.00	104.87–147.38 (9 samples)	90.25–121.71 (9 samples)
Long time sorption-moistened		
4.42	130.03–142.48 (4 samples)	–
f5.39	–	105.24–115.36 (4 samples)
Intensive short time sorption moistened		
15.17	107.62–117.43 (4 samples)	–
14.36	–	90.46–104.23 (4 samples)

**Table 2:** Properties of the materials used in simulations.

Properties	Hemp Shives	Flax Shives	Lime Plaster	Spruce	OSB	Clay Board	Gypsum Board
Thickness (m)	0.26	0.26	0.01	0.06 × 0.26	0.025	0.025	0.025
Density (kg/m^3^)	115.0	140.0	1249.0	414.6	595.0	1699.9	850.0
Porosity (m^3^/m^3^)	0.79	0.78	0.53	0.72	0.90	0.36	0.65
Specific heat (J/(kg·K))	1600.0	1400.0	999.0	2416.0	1500.0	833.8	850.0
Thermal conductivity of dry material (W/(m·K))	0.048	0.047	0.281	0.148	0.130	0.910	0.200
Diffusion resistance (–)	3.5	3.5	11.1	3.8	165.0	14.2	10.0

## Data Availability

The data presented in this study are available on request from the corresponding author. The data are not publicly available due to privacy.
